# Supplementary material for: Diagnostic potential of total serum ghrelin in autoimmune gastritis: A systematic review and meta-analysis
Source: PLoS One. 2026 Mar 12;21(3):e0344129. doi: 10.1371/journal.pone.0344129 (PMC12981498; doi:10.1371/journal.pone.0344129)
Supplement: S1 Table — (DOCX) [file pone.0344129.s001.docx]

**Table S1.** Search Strategy

| **Databases/Registers** | **Keywords** |
| --- | --- |
| PubMed | (((((((((((“Atrophic Gastritis”[Title/Abstract]) OR (“AG”[Title/Abstract])) OR (“Autoimmune Gastritis”[Title/Abstract])) OR (“AG”[Title/Abstract])) OR (“Autoimmune Atrophic Gastritis”[Title/Abstract])) OR (“AAG”[Title/Abstract])) OR (“Chronic Atrophic Gastritis”[Title/Abstract])) OR (“CAG”[Title/Abstract])) OR (“Chronic Atrophic Autoimmune Gastritis”[Title/Abstract])) AND (“Ghrelin”[Title/Abstract])) OR (“Serum Ghrelin”[Title/Abstract])) OR (“Total Serum Ghrelin”[Title/Abstract]) |
| EBSCO Host (in MEDLINE Full Text) | TI “Chronic Atrophic Gastritis” OR TI “Autoimmune Gastritis” OR TI “Autoimmune Atrophic Gastritis” OR TI “Chronic Atrophic Autoimmune Gastritis” AND TI “Ghrelin” OR TI “Serum Ghrelin” OR TI “Total Serum Ghrelin” |
| Scopus | TITLE-ABS-KEY ( “Atrophic Gastritis” ) OR TITLE-ABS-KEY (“AG” ) OR TITLE-ABS-KEY ( “Autoimmune Gastritis” ) OR TITLE-ABS-KEY (“AIG”) OR TITLE-ABS-KEY (“Autoimmune Atrophic Gastritis” ) OR TITLE-ABS-KEY (“AAG”) OR TITLE-ABS-KEY (“Chronic Atrophic Gastritis”) OR TITLE-ABS-KEY (“CAG”) OR TITLE-ABS-KEY (“Chronic Atrophic Autoimmune Gastritis”) AND TITLE-ABS-KEY (“Ghrelin”) OR TITLE-ABS-KEY (“Serum Ghrelin”) OR TITLE-ABS-KEY (“Total Serum Ghrelin”) |
| ProQuest | title(“Atrophic Gastritis”) OR title(“Autoimmune Gastritis”) OR title(“Autoimmune Atrophic Gastritis”) OR title(“Chronic Atrophic Gastritis”) OR title(“Chronic Atrophic Autoimmune Gastritis”) AND title(“Ghrelin”) OR title(“Serum Ghrelin”) OR “Total Serum Ghrelin” |
