# Supplementary figures and images for: Diagnostic potential of total serum ghrelin in autoimmune gastritis: A systematic review and meta-analysis
Source: PLoS One. 2026 Mar 12;21(3):e0344129. doi: 10.1371/journal.pone.0344129 (PMC12981498; doi:10.1371/journal.pone.0344129)

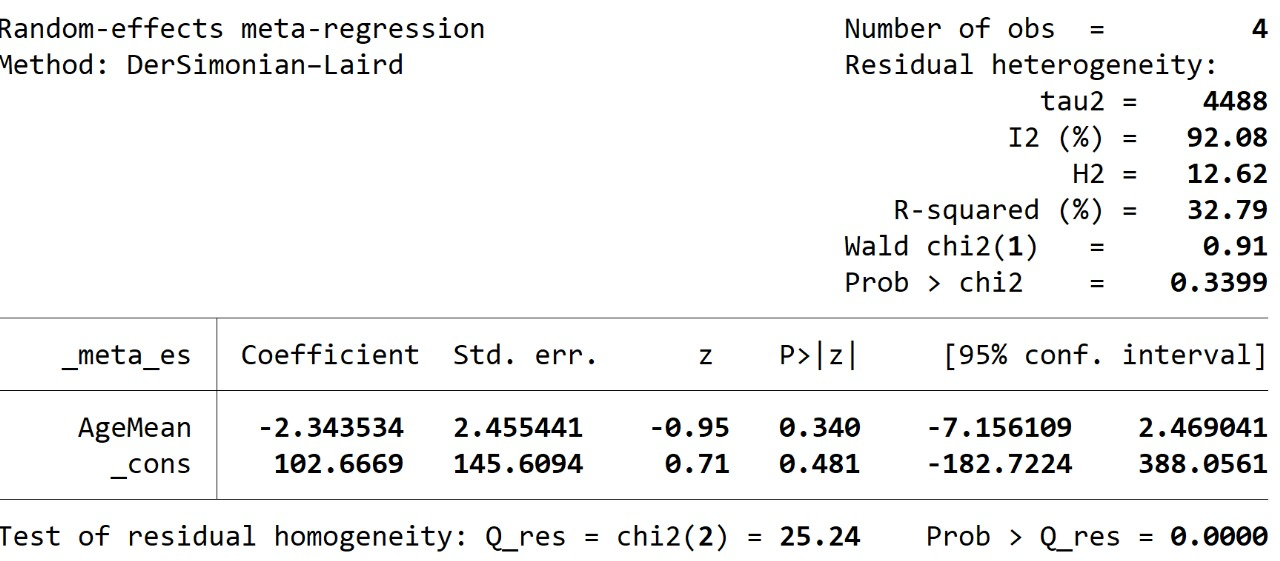
**Table S2.** Meta-regression Analysis of Mean Age Between Studies

Supplement: S2 Table — (DOCX) [file pone.0344129.s002.docx]

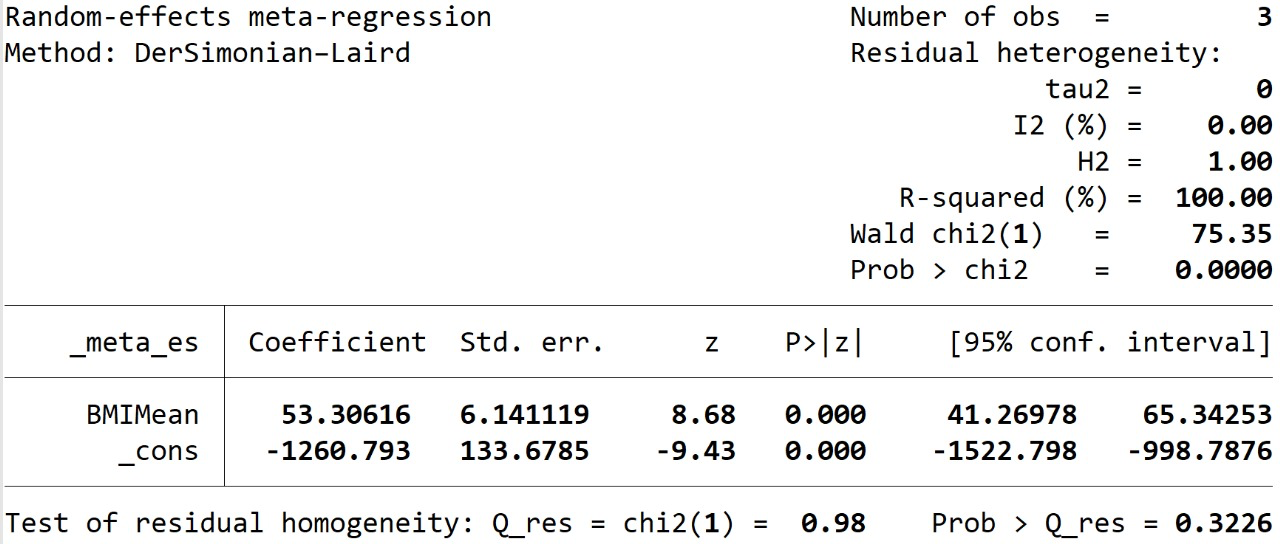
**Table S3.** Meta-regression Analysis of Mean BMI Between Studies

Supplement: S3 Table — (DOCX) [file pone.0344129.s003.docx]
