# Supplementary material for: Diagnostic potential of total serum ghrelin in autoimmune gastritis: A systematic review and meta-analysis
Source: PLoS One. 2026 Mar 12;21(3):e0344129. doi: 10.1371/journal.pone.0344129 (PMC12981498; doi:10.1371/journal.pone.0344129)
Supplement: S1 Fig — (DOCX) [file pone.0344129.s005.docx]

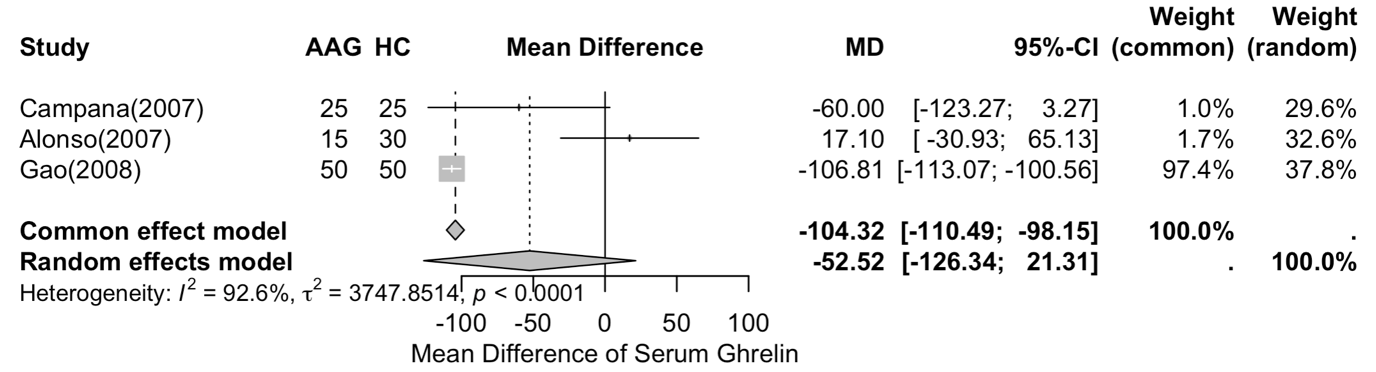
**Figure S1.** Sensitivity Analysis

The results showed the remaining non-significant difference in total serum ghrelin levels between AIG and healthy controls compared to the analysis with the inclusion of a study with a high risk of bias. The mean difference was -52.51 (95% CI: -126.34; 21.30), with very high heterogeneity (I^2^ = 92.6%).
