# Supplementary material for: Diagnostic potential of total serum ghrelin in autoimmune gastritis: A systematic review and meta-analysis
Source: PLoS One. 2026 Mar 12;21(3):e0344129. doi: 10.1371/journal.pone.0344129 (PMC12981498; doi:10.1371/journal.pone.0344129)
Supplement: Attachment — Submitted filename: Response to Reviewer.docx [file pone.0344129.s007.docx]

**Response to Reviewer/Editor’s Comment**

| Manuscript ID | : | PONE-D-25-40981 |
| --- | --- | --- |
| Title | : | **Diagnostic potential of total serum ghrelin in autoimmune gastritis: a systematic review and meta-analysis** |

**Responses to Editor**

1. Please ensure that your manuscript meets PLOS ONE's style requirements, including those for file naming. The PLOS ONE style templates can be found at https://journals.plos.org/plosone/s/file?id=wjVg/PLOSOne_formatting_sample_main_body.pdf and https://journals.plos.org/plosone/s/file?id=ba62/PLOSOne_formatting_sample_title_authors_affiliations.pdf

**Response:**

We have updated the manuscript formatting to comply with the PLOS ONE style requirements, including file naming and structural guidelines, as recommended.

2. We note that the grant information you provided in the ‘Funding Information’ and ‘Financial Disclosure’ sections do not match. When you resubmit, please ensure that you provide the correct grant numbers for the awards you received for your study in the ‘Funding Information’ section.

**Response:**

In the second submission, we ensure that the funding information and financial disclosure statements are fully aligned, including the correct grant numbers.

3. Please note that the funding information should not appear in any section or other areas of your manuscript. We will only publish funding information present in the Funding Statement section of the online submission form. Please remove any funding-related text from the manuscript.

**Response:**

We have removed all funding information from the manuscript files and will provide funding details exclusively in the **Funding Statement** section of the online submission form, as required.

4. Thank you for stating the following financial disclosure:

“This report is based on work supported in part by grants from the National Institutes of Health (DK62813) (Y.Y) and Grants-in-Aid for Scientific Research from the Ministry of Education, Culture, Sports, Science, and Technology (MEXT) of Japan (221S0002, 21H00346, 22H02871, and 23K24133) (Y.Y). This work was also supported by the Japan Agency for Medical Research and Development (AMED) [e-ASIA JRP; 23jm0210090h0003, Adopting Sustainable Partnerships for Innovative Research Ecosystem (ASPIRE); 23jf0126006h0001, Science and Technology Research Partnership for Sustainable Development (SATREPS); 23jm0110025h0003], and the Japan International Cooperation Agency (JICA) [SATREPS] (Y.Y). IT is a Ph.D. student supported by the Japanese Government (MEXT) Scholarship Program for 2024.”

Please state what role the funders took in the study.  If the funders had no role, please state: "The funders had no role in study design, data collection and analysis, decision to publish, or preparation of the manuscript." If this statement is not correct you must amend it as needed. Please include this amended Role of Funder statement in your cover letter; we will change the online submission form on your behalf.

**Response:**

The funders had no role in study design, data collection and analysis, decision to publish, or preparation of the manuscript. We will include this statement in the cover letter and ensure it is properly noted during the submission process.

5. Please include your tables as part of your main manuscript and remove the individual files. Please note that supplementary tables (should remain/ be uploaded) as separate "supporting information" files.

**Response:**

We have incorporated all tables into the main manuscript and prepared the supplementary tables as separate supporting information files, as requested.

6. Please include a copy of Table 2 which you refer to in your text on page 8.

**Response:**

We apologize for the confusion. The correct citations should be refer to Table 1, as the data have already been integrated into Table 1. We have corrected in the revised manuscript.

7. Please include captions for your Supporting Information files at the end of your manuscript, and update any in-text citations to match accordingly. Please see our Supporting Information guidelines for more information: http://journals.plos.org/plosone/s/supporting-information.

**Response:**

We have organized the **Supporting Information** by separating the figures and tables into individual files, and we have added the corresponding captions at the end of manuscript as required. All in-text citations have been updated accordingly.

8. If the reviewer comments include a recommendation to cite specific previously published works, please review and evaluate these publications to determine whether they are relevant and should be cited. There is no requirement to cite these works unless the editor has indicated otherwise.

**Response:**

Thank you for your advice and we followed your suggestion.

**Responses to Reviewers**

**Reviewer 1**

Reviewer #1: The manuscript is well written, and the topic is highly relevant, especially given the diagnostic challenges of autoimmune gastritis (AIG) and the growing interest in non-invasive serum biomarkers. However, there are significant methodological limitations that weaken the conclusions. The study included only five articles, two of which had a high risk of bias. Additionally, several points require improvement and more thorough discussion. Overall, this is a promising study on an important topic.

Major issues:

1. The authors include studies with AIG patients both with and without Helicobacter pylori infection. However, the introduction emphasizes that H. pylori confounds the clinical picture. This issue should be addressed more thoroughly by the authors throughout the discussion.

**Response:**

We have expanded the discussion to provide a more detailed explanation of the interaction between *H. pylori* infection and AIG. This additional context has been incorporated to address the concern raised.

*Several included studies were not excluding the patient with H. pylori infection. However, because the AIG occurrence may be associated with H. pylori infection (concurrently or post-eradication period), the result is still valid for interpretation. In addition, this result showed that the serum gherlin level may also associated with the condition of AIG and H. pylori infection that occurred concurrently. In the concurrent condition, the AIG was developed by molecular mimicry between H. pylori antigens and the H^+^/K^+^-ATPase with the invovelement of TLR-dependent pathway.[34] This was supported by recent finding in Indonesia that showed that the positivity of parietal cell antibody (PCA) is significantly correlated to the positivity of H. pylori infection. If the inflammation process persist after H. pylori eradication, the progression of AIG may occurred based on the gastric mucosal condition. However, the report of this condition is still limited to case reports.*

**Discussion, Line 213-223, Page 10**

2. The study's heterogeneity is very high, and the discussion of its relevance is insufficient. The result for the mean difference in total serum ghrelin between AIG patients and healthy controls was accompanied by high heterogeneity (I² = 98%). This level of I² indicates that the observed variation in the results was not random. All of this should be explored further in the discussion. Discuss in more detail the reasons for the high heterogeneity and what this implies for the results.

**Response:**

We acknowledge that the very high heterogeneity (I^2^ = 98%) limits the clinical interpretability of our findings. We have expanded the discussion to address this issue more thoroughly. Although we performed a meta-regression analysis, the limited availability of covariate data across the included studies allowed us to evaluate only two parameters. Nevertheless, we have added a detailed explanation of additional potential confounding factors.

*The high heterogeneity observed in the meta-analysis may be attributed to the small sample size and variations in participant demographics that may affect the effect size. A report by Levine T, et al. (2009) showed that the negative correlation between the sample size and effect size which lead to overestimation.[41] To explore potential sources of heterogeneity, a meta-regression analysis was conducted to the available possible confounding data: (1) age and (2) body mass index (BMI). The results indicated that mean BMI, but not mean age, was significantly associated with heterogeneity in pooled effect size (Tables S2 and S3). However, the other possible confounding factors may also contributed. Several report in Japan showed that plasma ghrelin level was inversely correlated with metabolic syndrome and or insulin resistance.[42–44] Female gender and high-density lipoprotein level were associated independently to the ghrelin level.[45] Another report showed that plasma ghrelin was positively correlated with serum albumin level.[46] In addition, the demographic status may also contribute. Matsunaga-Irie S, et al. (2007) reported that the serum ghrelin levels were significantly higher in Caucasian than Japanese that may cause by the higher prevalence of obesity among Caucasian than Japanese.[47] Unfortunately, the limitation of provided data was limiting the exploration of other confounding factors that cause the high heterogeneity.*

**Discussion, Line 237-252, Page 11**

3. The conclusions suggest the diagnostic utility of ghrelin in cases of moderate or severe gastric atrophy; however, this conclusion should be interpreted with greater caution. I recommend moderating the conclusion and highlighting the study's limitations. Although subgroup analysis provides valuable insights, the main conclusion should be based on the pooled outcome. Given the heterogeneity and bias in the analyses, the authors should exercise more caution and use more reserved language when interpreting the clinical significance of the subgroup findings, clearly stating that these findings lack robust validation.

**Response:**

We agree with your assessment that the limited number of studies and the high heterogeneity necessitate a more cautious interpretation of the findings. We have revised the conclusions to emphasize the restricted clinical applicability of this study and have moderated the interpretation of the subgroup analyses accordingly. We now explicitly acknowledge that these subgroup findings lack robust validation and should not be overgeneralized.

*The total serum ghrelin levels in patients with AIG were not significantly different from those in healthy controls. However, patients with AIG and moderate-to-severe gastric atrophy exhibited notably lower ghrelin levels. These findings suggest that total serum ghrelin level may serve as a potential diagnostic marker for AIG in patients with advanced gastric atrophy. However, given the limited number of subjects and included studies, the applicability of these findings in clinical practice remains restricted. Given the high heterogeneity, risk of bias, and other limitations among the included studies, further well-designed research is needed to validate the diagnostic utility of serum ghrelin for AIG.*

**Conclusion, Line 276-283, Page 12**

4. The authors state that they did not register the systematic review protocol in PROSPERO before data extraction. For current systematic reviews, the lack of registration is a methodological limitation that should be discussed rather than simply mentioned. Please include a section justifying the impact of this omission and discuss how methodological decisions were pre-defined.

**Response:**

We have expanded the *Registration and Protocol* section to clarify this point. We noted that the protocol was not registered in PROSPERO because the literature search and data extraction had already been initiated prior to protocol submission, and PROSPERO only accept registrations when the search has not yet begun. To ensure the methodological decisions, we conducted the systematic review based on PRISMA guideline.

*The study protocol was not registered with PROSPERO, as data synthesis had already commenced following a preliminary literature search. This study was conducted according to Preferred Reporting Items for Systematic reviews and Meta-analyses (PRISMA).*

**Registration and Protocol, Line 286-288, Page 13**

5. Although four databases are mentioned, there is inconsistency in the list of databases. The Methods section lists PubMed, EBSCO Host, Scopus, and ScienceDirect, while the Results section mentions PubMed, EBSCO Host, Scopus, and ProQuest. The authors should correct the list to accurately reflect the databases used.

**Response:**

We apologize for the inconsistency in reporting the databases used. We have corrected the *Methods* section to accurately reflect the databases searched: PubMed, EBSCO Host, Scopus, and ProQuest.

6. The search period is stated as "August 25 to September 31, 2024." September does not have 31 days, so this error should be corrected.

**Response:**

Thank you for pointing out this error. We have corrected the search period to August 25 to September 30, 2024 in the revised manuscript.

**Reviewer 2:**

Reviewer #2: Regarding the appreciated efforts of the authors in preparing this meta-analysis review by addressing the possible use of Ghrelin as indicative biomarker of AIG, there are some important notes should be discussed first:

Introduction section:

1. lines 86-89 lack the proper peer reviewed citations to support authors claims regarding AIG low incidence in Asian countries. Proper references can be: “5”, “Terao, S., Suzuki, S., Yaita, H., Kurahara, K., Shunto, J., Furuta, T., Maruyama, Y., Ito, M., Kamada, T., Aoki, R., Inoue, K., Manabe, N., & Haruma, K. (2020). Multicenter study of autoimmune gastritis in Japan: Clinical and endoscopic characteristics. Digestive endoscopy : official journal of the Japan Gastroenterological Endoscopy Society, 32(3), 364–372. https://doi.org/10.1111/den.13500“, and “Park JY, Cornish TC, Lam-Himlin D, Shi C, Montgomery E. Gastric lesions in patients with autoimmune metaplastic atrophic gastritis (AMAG) in a tertiary care setting. Am J Surg Pathol. 2010;34:1591–1598. doi: 10.1097/PAS.0b013e3181f623af.” “Notsu T, Adachi K, Mishiro T, et al. Prevalence of autoimmune gastritis in individuals undergoing medical checkups in Japan. Intern Med. 2019;58:1817–1823. doi: 10.2169/internalmedicine.2292-18.”

**Response:**

Thank you for this valuable suggestion. We acknowledge the need for appropriate peer-reviewed citations in the introduction. We have now updated the manuscript by incorporating two of the recommended references (Park JY, et al. and Notsu, et al.) to support the statement regarding the low incidence of AIG in Asian population. Additionally, we have included a recent meta-analysis on AIG to further strengthen the epidemiological context.

*Historically, the incidence of AIG in Asian countries was lower than in Western countries, as chronic atrophic gastritis in Asia has primarily been attributed to Helicobacter pylori infection. A report in Japan showed that from 6,739 subjects, there are 46 (0.49%) had the endoscopic appearance of AIG, but only 33 of 46 were diagnosed as AIG. Another report evaluates the AIG appearance in biopsy sample of several ethnicity, and the results showed that the AIG appearance only found in 1.4% of Asian ethnicity. A recent meta-analysis showed that the Europe, Africa, and Australia region were dominating the prevalence of AIG with the prevalence of 4.94%, 8.46%, and 8.08%. In comparison, the prevalence of AIG in Asia region is only 2.23%. However, recent studies suggest a shifting epidemiological trend, with increasing recognition of AIG among Asian populations.*

**Introduction, Line 32-41, Page 2**

2. Suggested references (lines 97-99):

- Park, J. Y., Cornish, T. C., Lam-Himlin, D., Shi, C., & Montgomery, E. (2010). Gastric lesions in patients with autoimmune metaplastic atrophic gastritis (AMAG) in a tertiary care setting. The American Journal of Surgical Pathology, 34(11), 1591–1598. <https://doi.org/10.1097/pas.0b013e3181f623af>
- Rugge, M., Bricca, L., Guzzinati, S., Sacchi, D., Pizzi, M., Savarino, E., Farinati, F., Zorzi, M., Fassan, M., Tos, A. P. D., Malfertheiner, P., Genta, R. M., & Graham, D. Y. (2022). Autoimmune gastritis: long-term natural history in naïveHelicobacter pylori-negative patients. Gut, 72(1), 30–38. <https://doi.org/10.1136/gutjnl-2022-327827>.
- Yu, Y., Tong, K., Shangguan, X., Yang, X., Wu, J., Hu, G., Yu, R., & Tan, C. (2023). Research status and hotspots of autoimmune gastritis: A bibliometric analysis. World Journal of Gastroenterology, 29(42), 5781–5799. <https://doi.org/10.3748/wjg.v29.i42.5781>
- Fuentes-Valenzuela, E., Cruz, S. E., Villanueva, J. P., Rodríguez, A. C., Díaz, A. G., Del Carmen López-Martín, M., De La Plaza, I. R., Blanco, S., Martín-Falquina, I. C., Aran, B. R., Martinez, R. L., Herrera, L. a. C., Olvera, R., Rodríguez, D. A., López, K. G., & Domínguez, A. B. (2025). Prevalence and evolution of newly diagnosed autoimmune gastritis in a large Spanish retrospective cohort. Revista Española De Enfermedades Digestivas, 117(8), 441–446. <https://doi.org/10.17235/reed.2025.11101/2025>

**Response:**

Thank you for the additional reference suggestions. We agree to include further supporting literature and have incorporated the study by Rugge M, et al., particularly to highlight the role of intrinsic factor autoantibodies in the diagnostic evaluation of AIG. The remaining references you suggested are consistent with the evidence we have already cited in the introduction.

3. Lines (103-106): Since this section describes the correlation between mucosal atrophy and declined serum ghrelin levels, the Inverse correlation between gastrin and histologic severity, and what are the functional implications that can be contributed with reduced ghrelin levels should be declared to support the authors’ hypothesis in Ghrelin use as diagnostic biomarker. (suggested references:

- Checchi, S., Montanaro, A., Pasqui, L., Ciuoli, C., Cevenini, G., Sestini, F., Fioravanti, C., & Pacini, F. (2007). Serum Ghrelin as a Marker of Atrophic Body Gastritis in Patients with Parietal Cell Antibodies. The Journal of Clinical Endocrinology & Metabolism, 92(11), 4346–4351. <https://doi.org/10.1210/jc.2007-0988>
- Kalkan, Ç., & Soykan, I. (2018). The relations among serum ghrelin, motilin and gastric emptying and autonomic function in autoimmune gastritis. The American Journal of the Medical Sciences, 355(5), 428–433. <https://doi.org/10.1016/j.amjms.2017.12.021>
- Wu W, Zhu L, Dou Z, Hou Q, Wang S, Yuan Z, Li B. Ghrelin in Focus: Dissecting Its Critical Roles in Gastrointestinal Pathologies and Therapies. Current Issues in Molecular Biology. 2024; 46(1):948-964. https://doi.org/10.3390/cimb46010061

**Response:**

We have revised the paragraph to better explain the molecular mechanisms underlying the inverse relationship between gastric mucosal pathology and circulating ghrelin levels, including the impact of parietal cell loss and endocrine dysfunction on ghrelin secretion. We have also expanded the discussion to clarify the functional implications of reduced ghrelin levels in the context of its proposed diagnostic utility. In addition, we agree with your suggestion and have incorporated the reference by Wu et al. (2024) to strengthen this section.

*Ghrelin is a potential biomarker of AIG. Ghrelin is a hormone secreted by endocrine cells in the oxyntic glands of the gastric corpus and the fundus, particularly by the P/D1 cells. This hormone has been reported to be associated with several conditions such as gastric cancer and colorectal cancer. In AIG, damage to the oxyntic mucosa or parietal cells at the fundus and corpus of the stomach may lead to progressive gastric mucosal atrophy. The atrophy may affect the level of ghrelin that predominantly produced in stomach.*

**Introduction, Line 55-60, Page 3**

4. Conceptual diagrams showing the relationship between AIG, ghrelin, and gastric atrophy to address the introduction concepts must be provided (by using visualization tools such as Canva or BioRender).

**Response:**

We have created a schematic diagram illustrating the relationship between AIG, ghrelin, and gastric atrophy, and this has been added as Figure 1 in the revised manuscript.

Methods

5. Search strategy: By referring to results section and supplementary table 2, the fourth database must be corrected as ProQuest and authors must pay attention to information integrity, consistency and accuracy prior to any future submission!

**Response:**

Thank you for pointing this out. We have corrected the fourth database to ProQuest in the Methods section to ensure consistency and accuracy across the manuscript.

6. Selection and Data Collection Process: I believe that Systematic Review Management is more appropriate to be for better handling of study selection and bias assessment (such as Covidence, Zotero or Rayyan), which can also be reflected in the discussion. Also, the avoidance of using any automated / AI tools for data collection should be explained and define the reasons behind applying the manual data collection instead!

**Response:**

Thank you for your thoughtful recommendation. While we acknowledge the value of systematic review management platforms such as Covidence, Zotero, and Rayyan, we opted to perform the study selection and data extraction manually. This decision was based on our experience conducting systematic review and meta-analyses, as well as guidance from established methodological references (e.g., Cohcrane Handbook and Introduction to Meta-Analysis by Borenstein et al.), which confirm that manual screening and extraction remain acceptable and methodologically sound.

We also chose the manual approach to allow for more careful and clinically informed decision-making by our reviewers during the selection process. Given that manual data collection is still aligned with the standard systematic review practices, we believed a detailed discussion of this choice is not necessary.

Results:

7. Study characteristics: This must be addressed in a summary (at least one to two paragraphs) along with the mentioned table 1; by illustrating the main parameters being mentioned, methods of Ghrelin measurement being used, and focus on 1/5th presentation of Asian countries among the included studies!

**Response:**

We have added a summary of the study characteristics in the Result section, accompanying Table 1. This summary highlights the key parameters of the included studies, the diagnostic methods for AIG, and the serum ghrelin measurement techniques used (ELISA and radioimmunoassay).

*The study’s characteristics are presented in Table 1. The five included studies comprised a total of 331 participants across Italy, Spain and China with sample sizes ranging from 15 to 100 subjects. All studies diagnosed AIG through a combination of endoscopic evaluation, histopathological confirmation, and APCA positivity, while methods for serum ghrelin measurement varied between ELISA and radioimmunoassay. Participant demographic differed across studies, reflecting differences in diagnostic methods, population characteristics, and sample handling. Key study limitations included lack of BMI stratification, incomplete H. pylori status reporting, absence of atrophy severity subgroup analyses, and potential confounding from comorbid conditions such as type 1 diabetes mellitus.*

**Result, Study Characteristics, Line 139-147, Page 7**

Discussion section:

8. The study includes a meta-analysis of five studies with subgroup analyses based on atrophy severity, enhancing the depth of interpretation. These analyses strengthen the robustness of the findings by exploring heterogeneity and bias sources and clarified the results transparency through well reporting of effect sizes, confidence intervals, and heterogeneity metrics (I², Q, H²). Despite meta-regression, the I² remains very high (>98%), and the discussion could better explore other sources (e.g., assay methods, geographic variation).

**Response:**

Thank you for your constructive suggestion. We have revised the Discussion section to further elaborate on the potential sources of the very high heterogeneity (I^2^ > 98%) observed in our meta-analysis. Specifically, we expanded the explanation as follows:

*The high heterogeneity observed in the meta-analysis may be attributed to the small sample size and variations in participant demographics. To explore potential sources of heterogeneity, a meta-regression analysis was conducted to the available possible confounding data: (1) age and (2) body mass index (BMI). The results indicated that mean BMI, but not mean age, was significantly associated with heterogeneity in pooled effect size (Tables S2 and S3). However, the other possible confounding factors may also be contributed. Several reports in Japan showed that plasma ghrelin level was inversely correlated with metabolic syndrome and or insulin resistance.[41–43] Another report showed that plasma ghrelin was positively correlated with serum albumin level.[44] In addition, the demographic status may also contribute. Matsunaga-Irie S, et al. (2007) reported that the serum ghrelin levels were significantly higher in Caucasian than Japanese that may cause by the higher prevalence of obesity among Caucasian than Japanese.[45] Unfortunately, the limitation of provided data was limiting the exploration of other confounding factors that cause the high heterogeneity.*

**Discussion, Line 237-252, Page 11**

9. As we’ve noticed before (in baseline characteristics table), that most studies are from Europe, with only one from Asia, limiting generalizability, and thus deep analysis should explain the studies limitation. Moreover, BMI is identified as a contributor to heterogeneity, but other potential confounders (e.g., age, comorbidities, medication use) are not explored.

**Response:**

We appreciate your insightful observation. We have expanded the Discussion to emphasize the limited geographic representation of the included studies. We acknowledge that BMI was identified as a significant contributor to heterogeneity; however, additional potential confounders could not be fully explored due to insufficient reporting across the included studies. We have clarified that these limitations in the available published data contained our ability to conduct deeper analyses of these factors.

*The high heterogeneity observed in the meta-analysis may be attributed to the small sample size and variations in participant demographics. To explore potential sources of heterogeneity, a meta-regression analysis was conducted to the available possible confounding data: (1) age and (2) body mass index (BMI). The results indicated that mean BMI, but not mean age, was significantly associated with heterogeneity in pooled effect size (Tables S2 and S3). However, the other possible confounding factors may also be contributed. Several report in Japan showed that plasma ghrelin level was inversely correlated with metabolic syndrome and or insulin resistance.[41–43] Female gender and high-density lipoprotein level were associated independently to the ghrelin level.[44] Another report showed that plasma ghrelin was positively correlated with serum albumin level.[45] In addition, the demographic status may also contribute. Matsunaga-Irie S, et al. (2007) reported that the serum ghrelin levels were significantly higher in Caucasian than Japanese that may cause by the higher prevalence of obesity among Caucasian than Japanese.[46] Unfortunately, the limitation of provided data was limiting the exploration of other confounding factors that cause the high heterogeneity.*

**Discussion, Line 237-252, Page 11**

10. The discussion acknowledges overreliance on small sample sizes but could better emphasize how it affects statistical power and interpretation.

**Response:**

We have expanded the Discussion to more clearly address the impact of small sample sizes on the reliability of our findings. Specifically, we have added an explanation noting that the limited number of participants reduces statistical power an may increase the risk of overestimating the true effect size. This limitation has been highlighted to ensure a more cautious interpretation of the results.

**Reviewer 3**

Reviewer #3: The study addresses a relevant clinical question regarding non-invasive diagnostics. However, the conclusions are heavily limited by the very high heterogeneity and the small number of available studies.

-Major Weaknesses and Limitations

1. Very High Heterogeneity: This is the most critical limitation of the meta-analysis. An `I²` value of 98% indicates that almost all the variation in results across studies is due to heterogeneity. The authors acknowledge this but the results should be interpreted with extreme caution.
2. Small Number of Included Studies: With only five studies, the analysis has limited statistical power.
3. The authors note that most studies were from Europe, with only one from Asia, and sample sizes were generally small. This limits the generalizability of the findings.

**Response:**

Dear Reviewer 3,

Thank you for your valuable feedback. We have expanded the Discussion section to more clearly address the impact of the very high heterogeneity (I^2^ = 98%) and the small number of included studies on the reliability of our findings. We now emphasize that these limitations substantially reduce statistical power and require cautious interpretation of the results. Additionally, we have highlighted that the predominance studies (from Europe) significantly limit the generalizability of our conclusions to broader population.

*The high heterogeneity observed in the meta-analysis may be attributed to the small sample size and variations in participant demographics that may affect the effect size. A report by Levine T, et al. (2009) showed that the negative correlation between the sample size and effect size which lead to overestimation.[41] To explore potential sources of heterogeneity, a meta-regression analysis was conducted to the available possible confounding data: (1) age and (2) body mass index (BMI). The results indicated that mean BMI, but not mean age, was significantly associated with heterogeneity in pooled effect size (Tables S2 and S3). However, the other possible confounding factors may also be contributed. Several reports in Japan showed that plasma ghrelin level was inversely correlated with metabolic syndrome and or insulin resistance.[42–44] Female gender and high-density lipoprotein level were associated independently to the ghrelin level.[45] Another report showed that plasma ghrelin was positively correlated with serum albumin level.[46] In addition, the demographic status may also contribute. Matsunaga-Irie S, et al. (2007) reported that the serum ghrelin levels were significantly higher in Caucasian than Japanese that may cause by the higher prevalence of obesity among Caucasian than Japanese.[47] Unfortunately, the limitation of provided data was limiting the exploration of other confounding factors that cause the high heterogeneity.*

**Discussion, Line 237-252, Page 11**

This study has several limitations. First, most of the included studies were conducted in Europe, with only one study from Asia (China) having a relatively small sample size that reduce its generalizability.

**Discussion, Line 253-255, Page 11**

The total serum ghrelin levels in patients with AIG were not significantly different from those in healthy controls. However, patients with AIG and moderate-to-severe gastric atrophy exhibited notably lower ghrelin levels. These findings suggest that total serum ghrelin level may serve as a potential diagnostic marker for AIG in patients with advanced gastric atrophy. However, given the limited number of subjects and included studies, the applicability of these findings in clinical practice remains restricted. Given the high heterogeneity, risk of bias, and other limitations among the included studies, further well-designed research is needed to validate the diagnostic utility of serum ghrelin for AIG.

**Conclusion, Line 276-283, Page 12**
